# Heteroployacid on the composite of boehmite and polyionic liquid as a catalyst for alcohol oxidation and tandem alcohol oxidation Knoevenagel condensation reactions

**DOI:** 10.1038/s41598-022-20699-2

**Published:** 2022-09-30

**Authors:** Neda Abedian-Dehaghani, Samahe Sadjadi, Majid M. Heravi

**Affiliations:** 1 Department of Chemistry, School of Physics and Chemistry, Alzahra University, Vanak, PO Box 1993891176, Tehran, Iran; 2 Gas Conversion Department, Faculty of Petrochemicals, Iran Polymer and Petrochemical Institute, PO Box 14975-112, Tehran, Iran

**Keywords:** Chemistry, Catalysis, Heterogeneous catalysis

## Abstract

Using boehmite as an available and low-cost natural compound, a bi-functional catalytic composite is prepared through vinyl-functionalization of boehmite, followed by polymerization with the as-prepared bis-vinylimidazolium bromide ionic liquid and supporting of phosphotungstic acid. The catalyst was characterized via ICP, XRD, TGA, FTIR, SEM/EDS and elemental mapping analysis and applied for promoting alcohol oxidation reaction and one-pot tandem alcohol oxidation/Knoevenagel condensation reaction in aqueous media under mild reaction condition. The results indicated high catalytic activity of the catalyst for both reactions. This protocol showed high generality and aliphatic, aromatic and heterocyclic alcohols could be applied as substrates to furnish the corresponding products in high to excellent yields. Furthermore, hot filtration test confirmed true heterogeneous nature of the catalysis. The catalyst could also be recovered readily and reused for at least five runs of the reaction with low loss of the activity and phosphotungstic acid leaching upon each run.

## Introduction

Nowadays, scientific research has been developed in response to the important challenges of green chemistry, such as designing of the chemical process with less synthetic steps in order to minimize the use of toxic solvents and reagents and waste production^1,2^. In this venue, planning the high-performance bi/multi-functional heterogeneous catalysts that can promote two or more reaction steps and can be easily separated from the reaction media is valuable^3^. Moreover, design of one-pot tandem reactions, which combine two or more synthetic steps in one-pot^4–6^ is an attractive approach for green synthesis of various chemicals^7,8^. As isolation of intermediates is not required in tandem reactions, they are also very appealing from economic point of view. In these reactions, mostly bi/multi-functional catalysts can be applied for promoting different steps of the reactions. One of the key tandem reactions is alcohol oxidation /Knoevenagel condensation reaction that is utilized for the synthesis of α,β-unsaturated nitriles as useful intermediates in organic synthesis. In this tandem reaction, alcohol is first oxidize to form the corresponding aldehyde, which then tolerates Knoevenoagel condensation with an active methylene compound to form the corresponding condensation product^3^. The appropriate catalyst for promoting this tandem reaction needs to possess both redox potential for catalyzing alcohol oxidation and acidic/basic characteristic for promoting Knoevenoagel condensation^9^. One of the most promising bi-functional catalyst with both redox and acidic features is heteropolycids, HPAs that are inorganic oxyacids of phosphorus and tungsten, molybdenum, vanadium, etc.^10^. Various types of HPAs, such as phosphotungstic acid have been extensively used for catalyzing both acid-catalyzed and oxidation reactions, such as epoxidation, alcohol oxidation etc.^11–15^. Some advantages of HPAs are their non-corrosive and non-toxic nature, while, their main inadequacy is their high solubility in common solvents, which caused problems in their recovery and recyclability procedures^12^. To circumvent this drawback, HPAs are mostly stabilized on supporting materials. Obviously, use of low-cost, available, bio-compatible and thermally stable supports is favoured for designing economic and environmentally benign heterogeneous catalysts. In this regard, boehmite is considered as an interesting candidate for heterogenizing various catalytic species^16^. Boehmite (γ-AlOOH) nanoparticles benefit from various advantageous, including stable structure, non-toxicity, high specific surface area, and availability^17^. In addition, the presence of hydroxyl groups on boehmite surface provides an opportunity to surface functionalization and tuning the properties of boehmite^18^. One of the attractive functional group for modifying supporting compounds, is ionic liquids (ILs) and their polymers (poly ionic liquids, PIL). ILs are organic salts, in which the cations are mostly heterocyclic compounds, such as imidazolium ion, and the anion can be conventional inorganic anions or even organic ones^19–21^. IL exhibits catalytic activity^22^ and can promote various organic transformations^23^. On the other hand, the charged nature of ILs can be exploited to provide electrostatic interactions with some catalytic species, such as HPAs. PILs are normally made by radical polymerization of IL monomers^24^ and as they contain multiple IL sites, they are considered not only as potential catalysts, but also as potent functional groups for modifying the supporting materials and improving stabilization of catalytic species.

**Figure 1:**
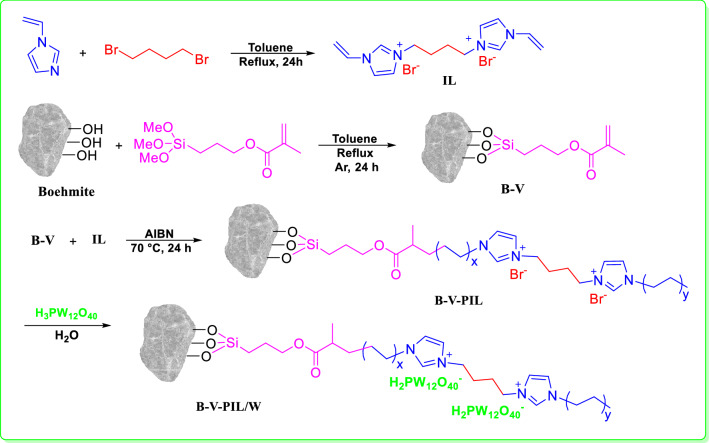
Pictorial synthetic route for the preparation of B-V-PIL/W.

Following our research on the utility of natural compounds for the preparation of heterogeneous catalysts^25–28^, we wish to introduce a novel catalytic composite, composed of PIL, phosphotungstic acid and boehmite. The catalyst was prepared through vinyl-functionalization of boehmite, followed by polymerization of IL and immobilization of phosphotungstic acid, Fig. 1. The catalytic performance of the catalyst was investigated for both alcohol oxidation and one-pot tandem alcohol oxidation /Knoevenagel condensation reaction in aqueous media.

## Result and discussion

### Structure of the catalyst

**Figure 2:**
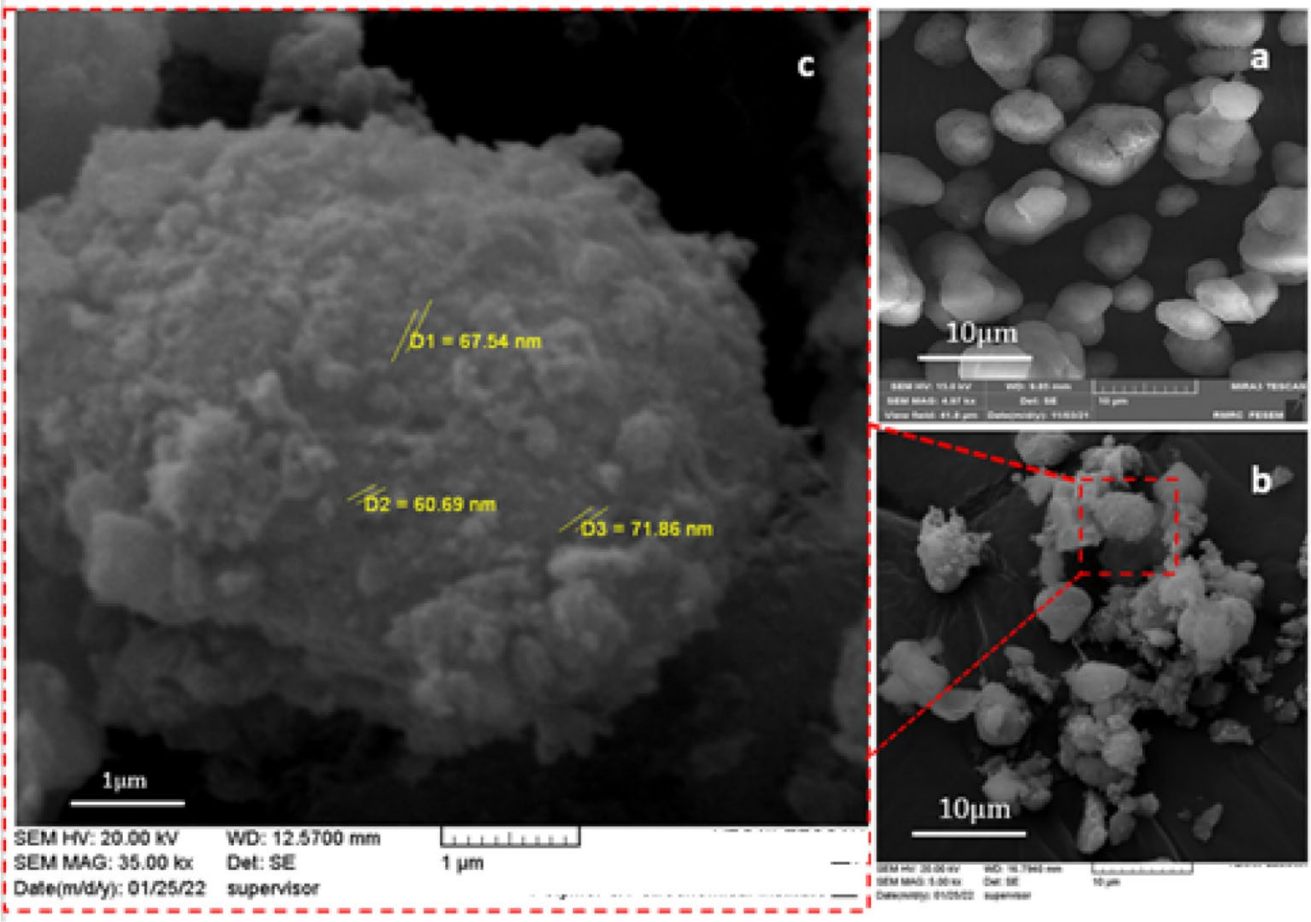
SEM images of (**a**) boehmite, (**b**, **c**) B-V-PIL/W.

The morphology of boehmite and B-V-PIL/W was examined by SEM analysis (Fig. 2). As shown in Fig. 2a, boehmite exhibited cubic orthorhombic morphology, while the morphology of B -V-PIL/W is distinguished. More precisely, in the SEM images of B-V-PIL/W, small aggregates covered boehmite orthorhombic cubes and rendered their surface rough (Fig. 2b,c).

**Figure 3:**
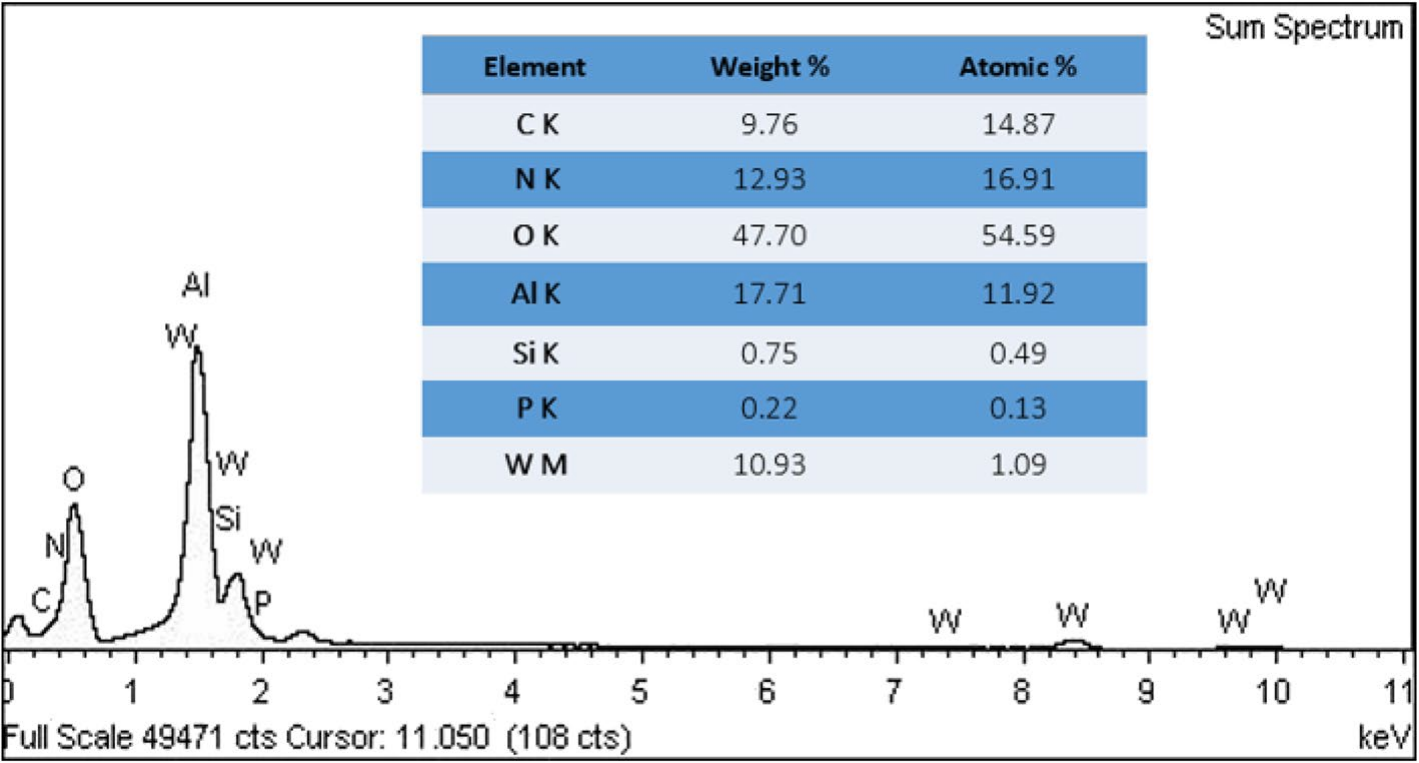
EDS analysis of B-V-PIL/W.

**Figure 4:**
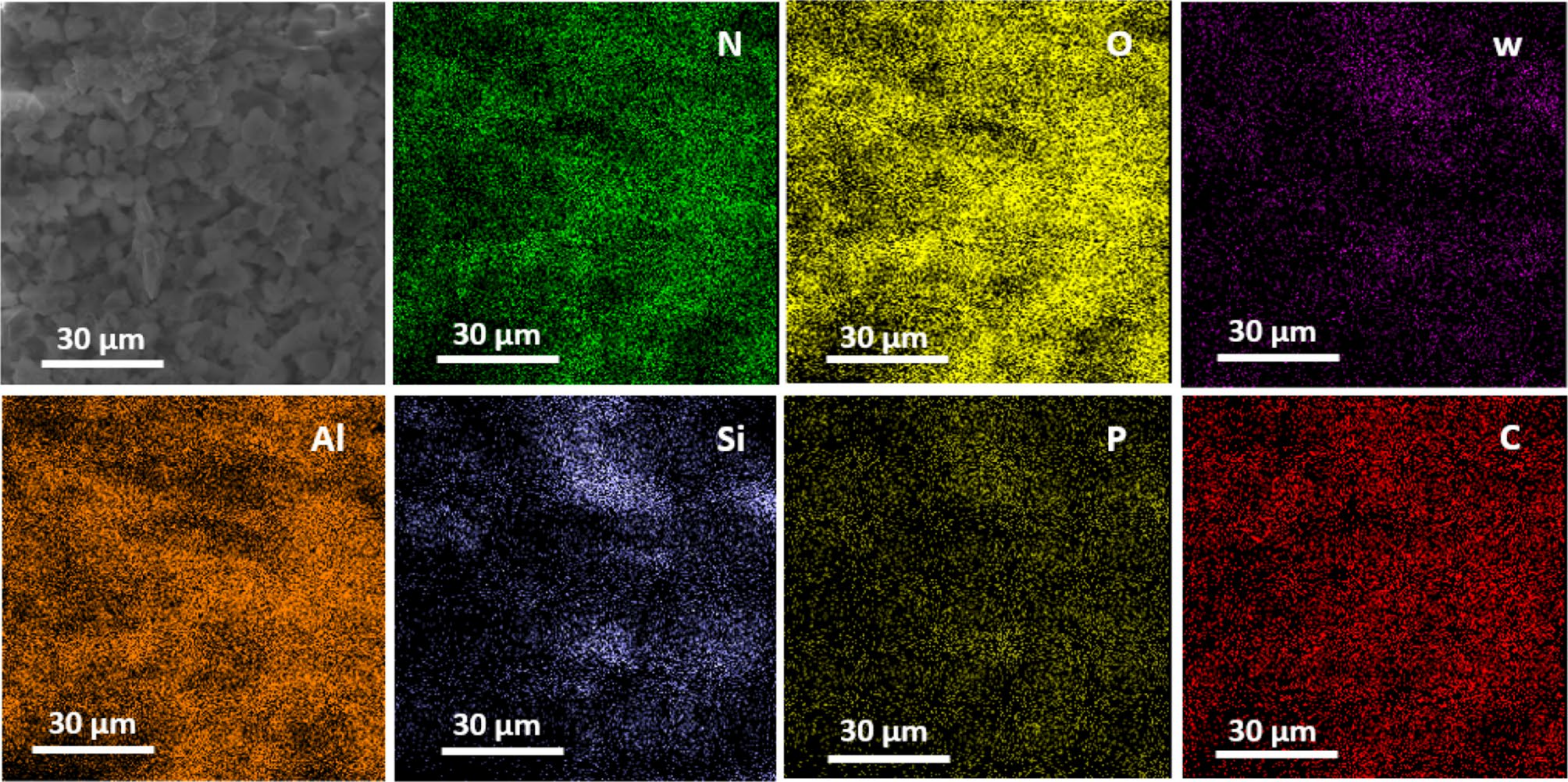
Elemental mapping of B-V-PIL/W.

EDS and elemental mapping techniques were used to prove the existence of the polymeric network and phosphotungstic acid on the catalyst. As shown in Fig. 3, EDS spectrum of B-V-PIL/W displays the presence of Al, O, C, Si, N, P, W. Among the detected elements, Al and O atoms are the elements in the framework of boehmite, while detection of C, O and Si atoms is indicative of conjugation of TMSPMA. The presence of PIL in the backbone of B-V-PIL/W can be proved by observation of C and N atoms and the existence of P, W and O atoms can imply the stabilization of phosphotungstic acid on the B-V-PIL and the preparation of the final catalyst (B-V-PIL/W) (Fig. 3). Figure 4 depicted the results of elemental mapping analysis of B-V-PIL/W. As shown, both C and N atoms that are representative of PIL exhibited high dispersion, indicating that PIL covered boehmite uniformly. Similarly, P and W atoms as the main atoms in phosphotungstic acid structure showed uniform dispersion, approving that phosphotungstic acid has been supported on B-V-PIL homogeneously.

**Figure 5:**
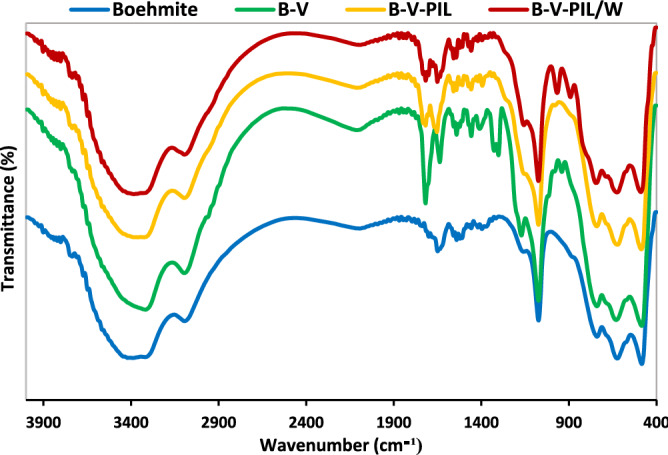
FTIR spectra of boehmite, B-V, B-V-PIL and B-V-PIL/W.

Figure 5 shows the FTIR spectra of boehmite, B-V, B-V-PIL and B-V-PIL/W. In the FTIR spectrum of boehmite, symmetric and asymmetric vibrations of the O–H bonds on the surface of boehmite are observed as strong and wide bands at 3080 and 3380 cm^−1^^29,30^. The absorbance bands at 480, 605 and 735 cm^−1^ are assigned to Al-O bands absorption^18^. Two strong bands at 1070 and 1161 cm^−1^ are related to the vibrations of hydrogen bonds of hydroxyl groups^31^. In the B-V spectrum, the presence of absorption bands at 1458 and 1718 cm^−1^ are related to the –C–O and –C=O vibrations, respectively, indicating the connection of TMSPMA to boehmite^32^. As well, the absorbance band appeared at 1630 cm^−1^ is related to axial deformation of C=C terminations^32^, while appearance of the bands at 2950, 1400, 1325, 1300 and 780 cm^−1^ are related to the symmetric, asymmetric and scissor-like stretching of the CH_2_ and CH_3_ groups^32^ in B-V respectively. In addition, the bands at 1045 and 930 cm^−1^ highlight the presence of Si–O–Si and Si–OH^32^. In the FTIR spectrum of B-V-PIL, all of the characteristic absorbance bands of B-V can be detected. Moreover, the band at 1665 cm^−1^ can be assigned to –C=N vibration^33^, which indicates the presence of PIL in the structure of the catalyst. In the FTIR spectrum of B-V-PIL/W, the bands at 1089 and 790 cm^−1^, which are assigned to the vibration of P–O and W–O–W bonds, respectively approve immobilization of H_3_PW_12_O_40_^34^.

**Figure 6:**
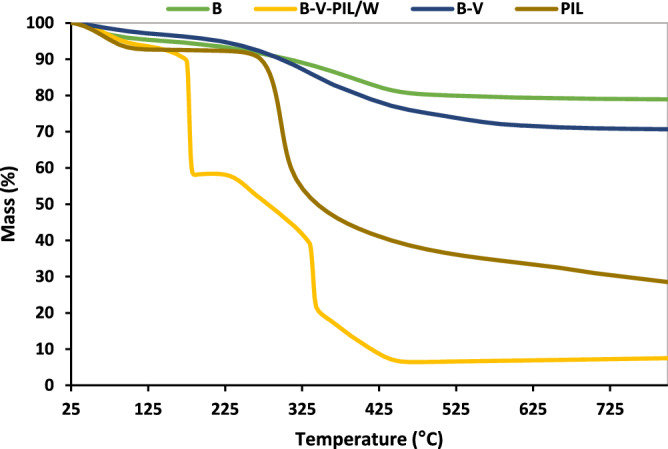
TG curves of boehmite, B-V, PIL and B-V-PIL/W.

The thermal stability of boehmite, B-V, PIL, and B-V-PIL/W was studied by TGA. As illustrated in Fig. 6, the initial weight loss observed at a temperature below 110 °C in all of the samples was caused by the removal of the absorbed water. Moreover, the weight loss at 450 °C is indicative of boehmite decomposition^29,35^. In the B-V thermogram an additional weight loss (10 wt%) was observed at 350 °C, which is related to the presence of V. PIL thermogram showed a weight loss about 325 °C that is due to the decomposition of PIL. In the thermogram of B-V-PIL/W, the weight loss, observed at 340 °C (23 wt%) is attributed to the decomposition of V-PIL moiety^36,37^.

**Figure 7:**
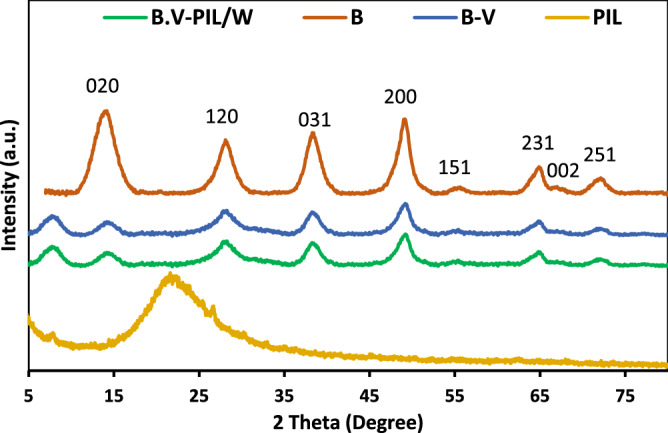
XRD patterns of boehmite (B), B-V, PIL, and B-V-PIL/W.

To appraise the effects of polymerization of PIL as well as H_3_PW_12_O_40_ immobilization on the structure of boehmite, XRD patterns of boehmite, B-V, PIL and B-V-PIL/W were compared, Fig. 7. As depicted, in the XRD pattern of boehmite, the characteristic peaks appeared at 2θ = 14.62°, 28.92°, 39.10°, 49.16°, 55.32°, 65.26° and 72.38°^35^. As shown, the XRD pattern of B-V was similar to that boehmit. This issue was quite expectable as the loaded content of V was low and as an organic functionality, it showed no peak in the XRD. PIL has an amorphous structure and shows a broad peak at 2θ = 15°–30°. XRD pattern of B-V-PIL/W is identical to that of boehmite and all of the characteristic peaks of boehmite can be detected in the XRD pattern of B-V-PIL/W with no shift and replacement.

ICP analysis of B-V-PIL/W affirmed that the content of H_3_PW_12_O_40_ on the catalyst was only 0.8 wt%.

### Catalyst activity

H_3_PW_12_O_40_ in the structure of B-V-PIL/W has both acidic features and redox potential. On the other hand, PIL is a potential catalyst for a wide range of reactions, such as Knoevenagel condensation. Hence, the catalytic activity of B-V-PIL/W was examined for two types of reactions. First, the performance of the catalyst was studied for oxidation of alcohols and then, tandem alcohol oxidation/Knoevenagel condensation reaction was carried out in the presence of B-V-PIL/W.

To investigate the performance of B-V-PIL/W for alcohol oxidation, oxidation of benzyl alcohol was selected as a model reaction and the reaction condition was optimized by altering the main variants (solvent, temperature and B-V-PIL/W loading). As summarized in Table 1, the model oxidation reaction was first conducted in water as solvent, H_2_O_2_ 30% (0.6 mmol) as oxidant and 10 mg B-V-PIL/W at 60 °C. As shown, under the aforementioned condition, the corresponding aldehyde was achieved in 30% yield. Next, H_2_O:EtOH (2:1) was applied as a solvent under the same reaction condition and it was found that altering the solvent could lead to improvement of the yield of the product. Subsequently, the effect of the catalyst amount was examined by using 10–50 mg B-V-PIL/W in H_2_O:EtOH (2:1) as solvent, H_2_O_2_ 30% (0.6 mmol) as oxidant at 60 °C. As tabulated, upon increase of the catalyst amount from 10 to 40 mg, the yield of the reaction increased and reached to 75%. It is worth mentioning that increment of the catalyst dosage to 50 mg was not effective. Finally, the effect of the reaction temperature was appraised and it was confirmed that upon increase of this value to 70 °C, the reaction yield enhanced to 95%. Noteworthy, further increase of the reaction temperature led to the decrease of yield of the desired product (benzaldehyde) due to the formation of benzoic acid as a by-product. Considering these experiments, the optimum reaction condition for alcohol oxidation was using 40 mg B-V-PIL/W in H_2_O:EtOH (2:1) as solvent, H_2_O_2_ 30% (0.6 mmol) as oxidant at 70 °C.

**Table 1:** Optimization of the reaction condition for the alcohol oxidation.

Entry	B-V-PIL/W (mg)	Solvent	Temp. (°C)	Yield (%)
1	10	H_2_O	60	30
2	10	H_2_O:EtOH (2:1)	60	40
3	20	H_2_O:EtOH (2:1)	60	50
4	30	H_2_O:EtOH (2:1)	60	60
5	40	H_2_O:EtOH (2:1)	60	75
6	50	H_2_O:EtOH (2:1)	60	75
7	40	H_2_O:EtOH (2:1)	70	95
8	40	H_2_O:EtOH (2:1)	80	90

The generality of alcohol oxidation reaction under the optimum condition was confirmed by using several alcohols with different steric and electronic features, Table 2. As listed, oxidation of aromatic and heterocyclic substrates was more efficient than aliphatic ones. Furthermore, the presence of electron-withdrawing functional groups on the aromatic ring led to the higher yield of the product.

**Table 2:** Oxidation reaction of various alcohols catalyzed by B-V-PIL/W.


Entry	Substrate	Product	Time (min)	Yield (%)^a^	TON^b^	TOF^c^ (h^−1^)
1			70	95 ± 1	8550	7307
2			45	97 ± 2	8730	11,640
3			65	90 ± 1	8100	7479
4			60	90 ± 2	8100	8100
5			70	92 ± 2	8280	7137
6			90	90 ± 2	8100	5400
7			90	90 ± 1	8100	5400
8			120	89 ± 1	8010	4005
9			150	80 ± 2	7200	2880
10			150	75 ± 1	6750	2700
11			150	75 ± 2	6750	2700
12			130	78 ± 2	7020	3235
13			120	78 ± 1	7020	3510
14			150	75 ± 2	6750	2700
15			160	75 ± 2	6750	2537

Reaction condition: alcohol (1 mmol), H_2_O_2_ 30% (1 mmol), B-V-PIL/W 40 mg in H_2_O/EtOH (2/1) at 70 °C.

^a^Isolated yield.

^b^$$\text{TON}=\frac{\text{Mol of produced aldehyde }}{\text{Mol of HPA on the catalyst}}$$.

^c^$$\text{TOF}=\frac{\text{TON}}{\text{Time }(\text{h})}$$^36,37^.

The results of alcohol oxidation reaction confirmed that B-V-PIL/W could effectively oxidize various alcohols. Motivated by those results, it was also examined whether B-V-PIL/W could act as a bi-functional catalyst and promote both alcohol oxidation reaction and Knoevenagel condensation in one-pot step. In fact, it was assumed that redox potential of H_3_PW_12_O_40_ could catalyze alcohol oxidation step, while PIL in the backbone of B-V-PIL/W as well as H_3_PW_12_O_40_ as an acidic catalyst could promote Knoevenagel condensation. To this purpose, tandem alcohol oxidation /Knoevenagel condensation of benzyl alcohol and malononitrile was selected as a model reaction and conducted under the aforesaid reaction condition. Gratifyingly, it was found that under the optimum reaction condition, the desired product was furnished in 94% yield, Table 3. Notably, not only benzyl alcohol could tolerate the one-pot tandem alcohol oxidation /Knoevenagel condensation reaction, but also various alcohols with different characteristic could be applied to furnish good to excellent yields of the corresponding products, Table 3, affirming the generality of this protocol.

**Table 3:** One-pot tandem oxidation /Knoevenagel condensation of alcohols with malononitrile catalyzed by B-V-PIL/W.


Entry	Substrate	Product	Time (min)	Yield (%)^a^	TON^b^	TOF^c^ (h^−1^)
1			90	94 ± 2	8460	5640
2			65	97 ± 1	8730	8083
3			65	98 ± 1	8820	8167
4			65	90 ± 2	8100	7500
5			90	92 ± 1	8280	5520
6			90	92 ± 2	8280	5520
7			90	92 ± 1	8280	5520
8			120	90 ± 2	8100	4050
9			120	90 ± 2	8100	4050
10			180	80 ± 2	7200	2400
11			185	80 ± 2	7200	2337
12			185	80 ± 1	7200	2337
13			180	80 ± 2	7200	2400
14			160	83 ± 1	7470	3626
15			150	85 ± 2	7650	5100
16			180	80 ± 2	7200	2400
17			180	80 ± 1	7200	2400

Reaction condition: alcohol (1 mmol), malononitrile (1.2 mmol), H_2_O_2_ 30% (1 mmol), B-V-PIL/W 40 mg in H_2_O/EtOH (2/1) at 70 °C.

^a^Isolated yield.

^b^$$\text{TON}=\frac{\text{mol of knoevenagel product}}{\text{mole of HPA on the catalyst}}$$.

^c^$$\text{TOF}=\frac{\text{TON}}{\text{Time }(\text{h})}$$^36,37^.

### Recyclability

**Figure 8:**
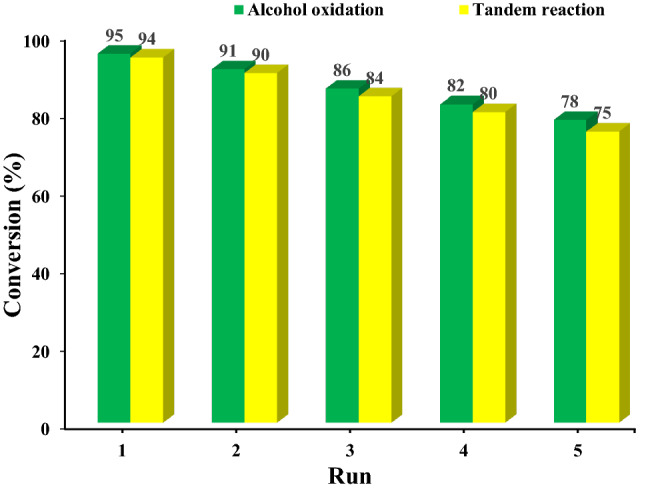
Recyclability of B-V-PIL/W for the model reactions under the optimum reaction condition.

The results of the catalytic activity approved high activity of B-V-PIL/W for both alcohol oxidation and one-pot tandem alcohol oxidation/Knoevenagel condensation reaction. As recyclability of a catalyst is also an important factor for large-scale uses, the recyclability of B-V-PIL/W for both aforementioned reactions was also investigated. To this purpose, oxidation of benzyl alcohol was selected as a model reaction and separated B-V-PIL/W after the end of the first run of the reaction, was recovered (see the “Experimental section” for more detail) and reused for the next run under the exactly identical reaction condition. As displayed in Fig. 8, recycling of B-V-PIL/W led to slight loss of the catalytic activity after each reaction run for both targeted reactions.

**Figure 9:**
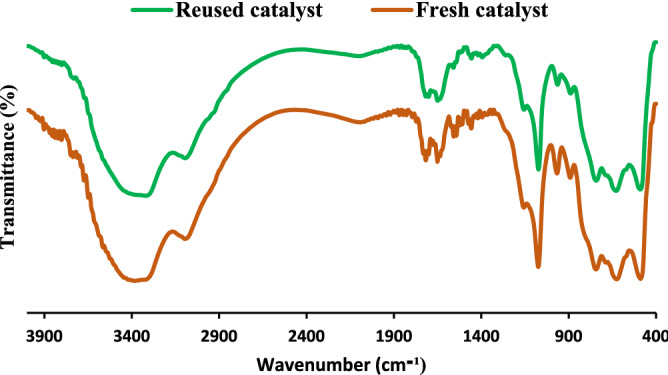
Comparison of the FTIR spectra of fresh and reused B-V-PIL/W after 5 runs of the model alcohol oxidation reaction.

Considering high solubility of H_3_PW_12_O_40_ in aqueous media, it was postulated that the observed loss of the activity can be related to the low leaching of H_3_PW_12_O_40_. To verify this assumption, ICP analysis was conducted for the catalyst recovered from the last run of the reaction and the result of this analysis revealed slight leaching of H_3_PW_12_O_40_ (1 wt% of initial dosage). The stability of B-V-PIL/W upon recycling was also appraised by comparing the FTIR spectrum of fresh and recycled B-V-PIL/W after 5 runs of the model alcohol oxidation reaction, Fig. 9. The similarity of the two FTIR spectra is a proof for the structural stability of B-V-PIL/W upon recycling.

### Control catalysts

In the next step, the catalytic activity of B-V-PIL/W for the oxidation of benzyl alcohol as the model reaction was compared with that of some control catalysts, including HPA, B/W, PIL/W and PIL/W, Table 4, to show the merit of hybridization of B and PIL. As shown, HPA showed high catalytic activity for the model oxidation reaction. However, its main drawback is its high solubility and consequently its tedious recovery and reuse. Regarding PIL/W, high catalytic activity was observed that can be due to the role of PIL in the catalysis. As shown, the activity of B/W was lower than that of PIL/W, which further implied the role of PIL in promoting the reaction. Comparison of the catalytic activity of B/W and PIL/W, it can be observed that their catalytic activity was lower than that of B-V-PIL/W, indicating the synergism between B and PIL.

**Table 4:** Comparison of catalytic activities of B-V-PIL/W and control catalysts for the model oxidation reaction.

Entry	Catalyst	Yield (%)^b^
1	HPA^a^	96
2	B/W	67
3	PIL/W	80
4	B-V-PIL/W	95

^a^Oxidation reaction condition: benzyl alcohol (1 mmol), H_2_O_2_ 30% (1 mmol), Catalyst (40 mg) in H_2_O/EtOH (2/1) at 70 °C, HPA (H_3_PW_12_O_40_).

^b^Isolated yield.

### Hot filtration

**Figure 10:**
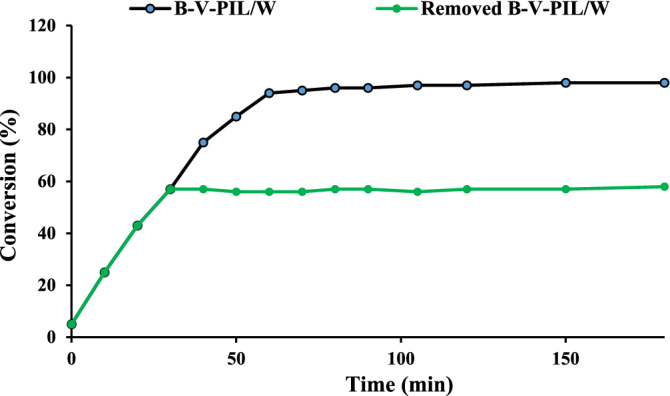
The result of hot filtration test for the model alcohol oxidation reaction under optimum reaction condition.

The nature of catalysis in alcohol oxidation reaction under B-V-PIL/W catalysis was studied via hot filtration test^38^. According to the standard protocol, B-V-PIL/W was removed from the reaction media after short reaction time and the reaction was monitored after catalyst removal to check whether it can progress in the absence of the catalyst. As the result showed, Fig. 10, upon removal of B-V-PIL/W, no progress was observed and the yield of the reaction did not change, confirming that the catalysis was true heterogeneous.

### Comparative study

The catalytic activity of B-V-PIL/W for tandem alcohol oxidation/Knoevenagel condensation reaction of benzyl alcohol was compared with some other catalysts. The summary of the reaction conditions and yields of the product indicate that various catalysts have been developed for this key reaction. Notably, different reaction conditions make precise comparison impossible, however, in some of the reported procedures, the reaction time was very long. Furthermore, from the data listed in Table 5 it can be concluded that some catalyst, such as NH_2_-UiO-66(Zr), NH_2_-MIL-101(Fe) and NH_2_-MIL-125(Ti) led to relatively low yields. Regarding other catalysts, comparable yields were obtained, indicating that B-V-PIL/W can be considered as an efficient catalyst that can catalyze tandem alcohol oxidation /Knoevenagel condensation reaction under environmentally friendly condition in relatively short reaction time.

**Table 5:** Comparison of the activity of B-V-PIL/W for one-pot tandem alcohol oxidation/Knoevenagel condensation reaction with some reported catalysts.

Entry	Catalyst	Time (h)	Catalyst amount	Condition	Yield (%)	Ref.
1	B-V-PIL/W	1.5	40 mg	H_2_O_2_/H_2_O:EtOH (2:1)/70 °C	94	This work
2	NH_2_-UiO-66(Zr)	40	20 mg	Trifluorotoluene: CH_3_CN/O_2_/light irradiation	4.6	^39^
3	NH_2_-MIL-101(Fe)	40	20 mg	Trifluorotoluene: CH_3_CN/O_2_/light irradiation	72	^39^
4	Ti-MOF-NH_2_	48	100 mg	*p*-Xylene/UV irradiation	32	^40^
5	Zr-MOF-NH_2_	48	100 mg	*p*-Xylene/UV irradiation	91	^40^
6	UoB-2^a^	1.5	2 mol%	Alcohol oxidation in solvent-free condition/TBHP/Knoevenagel condensation in EtOH	94	^38^
7	Cu_3_TATAT-3^b^	12	8 mol%	CH_3_CN/O_2_/75 °C	95	^41^
8	Fe_3_O_4_@SiO_2_@PEI@Ru(OH)_X_	22	100 mg	O_2_/110 °C for alcohol oxidation step/Knoevenagel condensation step at r.t.	90.2	^42^
9	NH_2_-MIL-125(Ti)	40	20 mg	Trifluorotoluene: CH_3_CN/O_2_/light irradiation	3.3	^39^
10	MNP@PIL/W^c^	6^d^	4 mol%	Alcohol oxidation in H_2_O_2_, H_2_O, 90 °C/Knoevenagel condensation step at r.t.	94	^43^

^a^Ni‐based metal–organic framework.

^b^H_6_TATAT = 5,5,5-(1,3,5-Triazine-2,4,6 triyl)tris(azanediyl)triisophtalate.

^c^Tungstate-loaded triazine-based magnetic Poly(Bis-imidazolium ionic liquid).

^d^Oxidation step at 90 °C (4 h) + Knoevenagel condensation step at room temperature (2 h).

### Reaction mechanism

**Figure 11:**
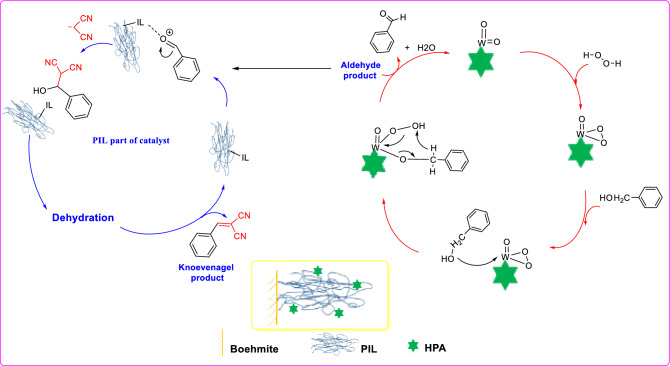
The proposed mechanism for tandem alcohol oxidation/Knoevenagel condensation reaction.

According to the literature, in the first step of the reaction, H_2_O_2_ binds to the transitional metal atom of HPA (W) and then one proton of H_2_O_2_ is transferred to one of the oxygen atoms in HPA to form HO_2_^−^, which is responsible for the oxidation of alcohol and formation of aldehyde and water^44,45^. Then, the ionic liquids in the structure of the catalyst as well as HPA can promote Knoevenagel reaction through activation of aldehyde, Fig. 11.

## Experimental section

### Materials and instruments

The chemicals used for the synthesis of the catalyst and conducting the tandem alcohol oxidation/Knoevenagel condensation reaction are as follow: boehmite, 3-(trimethoxysilyl) propyl methacrylate (98%, TMSPMA), 1,4-dibromobutane (99%), 1-vinylimidazole (≥ 99%), toluene (99.8%), diethyl ether (≥ 99.7%), sodium chloride (≥ 99%), magnesium sulfate (≥ 99.5%, MgSO_4_), ethyl acetate (99.8%), n-hexane (95%), ethanol (95%, EtOH), azobisisobutyronitrile (98%, AIBN), phosphotungstic acid (reagent grade, H_3_PW_12_O_40_), malononitrile (≥ 99%), hydrogen peroxide (30% (w/w) in H_2_O) and alcohol derivatives including benzyl alcohol (99.8%), 2-nitrobenzyl alcohol (97%), 4-nitrobenzyl alcohol (99%), 4-methyl benzyl alcohol (98%), 4-chloro benzyl alcohol (99%), 4-hydroxy benzyl alcohol (99%), 4-aminobenzyl alcohol (98%), furfuryl alcohol (synthesis grade), 4-methoxy benzyl alcohol (98%), 2-phenyl ethanol (synthesis grade), 3-methylbutanol (98.5%), 1-heptanol (98%), 1-pentanol (99%), 1-butanole (99.8%), 1-propanol (≥ 99.9%), isoamyl alcohol (98%), 2-methy-l-propanol (99.5%), all were purchased from Sigma-Aldrich (Germany, Taufkirchen).

The characterization techniques applied to verify formation of the catalyst, B-V-PIL/W, were as follow: Fourier transform Infrared spectroscopy (FT-IR), Inductively Coupled Plasma (ICP), X-ray diffraction (XRD), thermogravimetric analysis (TGA), Scanning electron microscopy (SEM) and Energy-Dispersive X-ray analysis (EDS), Nuclear magnetic resonance (NMR) spectroscopy.

All FTIR spectra were recorded using BRUKER TENSOR 35 spectrophotometer 65 (Germany) using KBr pellet. The scanning time of and spectral resolution were 1 s and 2 cm^−1^ respectively. The XRD pattern of boehmite and the prepared B-V-PIL/W were obtained using a Rigaku Ultima IV instrument (Japan) with Cu Kα radiation from a sealed tube. In order to study the thermal stability of B-V-PIL/W, METTLER TOLEDO apparatus (UK) was used. The thermogram was recorded under O_2_ atmosphere and heating rate of 10 °C min^−1^. SEM/EDS and elemental mapping analyses were performed on MIRA 3 TESCAN-XMU (Czech Republic). Vista-pro device (Australia) was used for conducting ICP analysis. The ^1^HNMR and ^13^CNMR spectra were obtained using BRUKER 400 MHz UltraShield (Germany).

### Preparation of the catalyst: B-V-PIL/W

#### Synthesis of bis-vinylimidazolium bromide ionic liquid: IL

Bis-vinylimidazolium bromide ionic liquid was synthesized according to the previously reported method^46^. In a typical procedure, a solution of 1-vinylimidazole (20 mmol, 1.8 mL) in toluene was prepared, mixed with 1,4-dibromobutane (10 mmol, 1.2 mL) and refluxed for 24 h. Then, the obtained product was filtered, washed with diethyl ether 3 times and dried at ambient temperature.

#### Functionalization of boehmite with TMSPMA: synthesis of B-V

In order to conjugate the as-prepared IL to boehmite, boehmite was first functionalized with TMSPMA. To this purpose, boehmite (2.5 g) was dispersed in dry toluene and then mixed with TMSPMA (8 mmol, 2.09 g). The mixture was refluxed under Ar atmosphere for 24 h to furnish vinyl-functionalized boehmite, B-V. Finally, the product was filtered, washed with toluene and dried in oven at 60 °C.

#### Conjugation of PIL to the functionalized boehmite: synthesis of B-V-PIL

First, a mixture of B-V (2 g) in EtOH/H_2_O (1/1, 30 mL) was prepared in a round bottle flask. Then, the as-prepared IL (4 mmol, 1.6 g) was dissolved in EtOH/H_2_O (1/1, 50 mL) and added to the B-V mixture and stirred for 0.5 h at 70 °C under Ar atmosphere. Subsequently, a solution of AIBN [0.3 g in EtOH (5 mL)] was added in a dropwise manner to the previous mixture for initiating the polymerization reaction. Polymerization was continued for 24 h at 70 °C and then the reaction vessel was cooled and the product was collected, washed with EtOH and H_2_O and dried at 70 °C overnight.

#### Immobilization of HPA on B-V-PIL: synthesis of B-V-PIL/W

To immobilize phosphotungstic acid on B-V-PIL, the incipient wetness impregnation method was used^47^. Typically, a 20% w/w solution of H_3_PW_12_O_40_ (0.32 g of HPA in 10 mL deionized water) was prepared and added to a suspension of the B-V-PIL (1.6 g) in deionized water (25 mL) in a dropwise manner. The mixture was then stirred for 24 h at room temperature and at the end, the precipitate was filtered, rinsed with deionized water (15 mL) and dried at 70 °C for 24 h, Fig. 1.

### Oxidation of alcohol

Oxidation of alcohols was conducted as follow: In a round bottle flask, a mixture of alcohol (1 mmol), H_2_O_2_ 30% (0.6 mmol), B-V-PIL/W (40 mg) in H_2_O:EtOH (2:1) (3 mL) was added and the resultant mixture was stirred at 80 °C. Using TLC, the progress of the reaction was followed and at the end of the reaction, the catalyst was separated, washed with EtOH and dried in an oven at 80 °C. The recovered catalyst was used for the next run of the oxidation reaction and the study of the recyclability of the catalyst. To achieve the oxidation product, diethyl ether (14 mL) was added to the filtrate and the mixture was washed with brine (3_˟_8 mL) and dried by MgSO_4_. Then, it was filtered and the solvent was evaporated under reduced pressure to obtain the crude product. To provide the pure aldehydes, column chromatography using ethyl acetate: n-hexane (1:9) as the eluent was used.

### General procedure for one-pot tandem oxidation/Knoevenagel condensation

For the one-pot tandem reactions, a mixture of alcohol (1 mmol), malononitrile (1.2 mmol), H_2_O_2_ 30% (0.6 mmol), B-V-PIL/W (40 mg) in H_2_O/EtOH (2/1) (3 mL) was transferred to a round bottle flask equipped with a reflux condenser and the mixture was stirred at 80 °C. The progress of the reaction was monitored by TLC and after completion of the process, B-V-PIL/W was filtered and recovered by washing with EtOH and drying at 70 °C. The recovered catalyst was used for the next run of the tandem reaction and the recyclability of the catalyst was studied. The resultant product was attained by evaporating of the solvent and purification via column chromatography, Figs. S1–S10. Using GC analysis, the yields of the reactions were calculated.

## Conclusion

A triple composite, composite of PIL, boehmite and phosphotungstic acid is designed and prepared through vinyl-functionalization of boehmite, followed by polymerization with the as-prepared bis-vinylimidazolium bromide IL and immobilization of phosphotungstic acid. The catalyst, B-V-PIL/W, was characterized and then applied for catalyzing both alcohol oxidation reaction and one-pot tandem alcohol oxidation /Knoevenagel condensation reaction in aqueous media under mild reaction condition. Gratifyingly, the results confirmed high catalytic activity of the catalyst that was comparable or superior to some of the previously reported catalysts. Notably, this protocol could be generalized to various alcohols with different steric and electronic features. Moreover, the recovered B-V-PIL/W could be successfully recycled and only slight loss of the catalytic activity and phosphotungstic acid leaching were observed upon each run of recycling. Hot filtration test also affirmed heterogeneous nature of the catalysis.

## Supplementary Information

Supplementary Figures.

## Data Availability

The datasets used and/or analyzed during the current study are available from the corresponding author on reasonable request.
