# Supplementary material for: Heteroployacid on the composite of boehmite and polyionic liquid as a catalyst for alcohol oxidation and tandem alcohol oxidation Knoevenagel condensation reactions
Source: Sci Rep. 2022 Sep 30;12:16395. doi: 10.1038/s41598-022-20699-2 (PMC9525677; doi:10.1038/s41598-022-20699-2)
Supplement: Supplementary file 1 — Supplementary Figures. [file 41598_2022_20699_MOESM1_ESM.docx]

**Supplementary information**

**Heteroployacid on the composite of boehmite and polyionic liquid as a catalyst for alcohol oxidation and tandem alcohol oxidation Knoevenagel condensation reactions**

Neda Abedian-Dehaghani^1^, Samahe Sadjadi^2*^, Majid M. Heravi^*1^


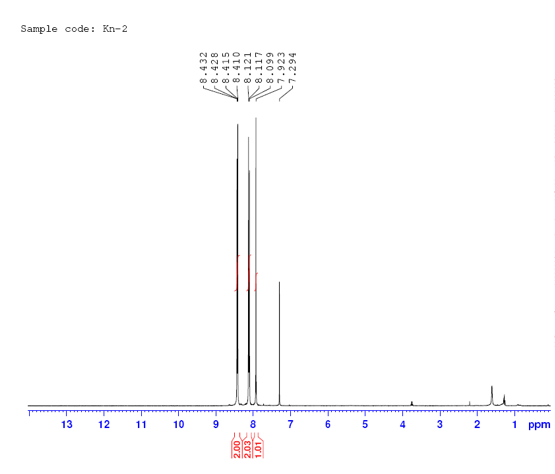


Figure S1. ^1^HNMR spectrum of 2-(4-nitrobenzylidene)malononitrile


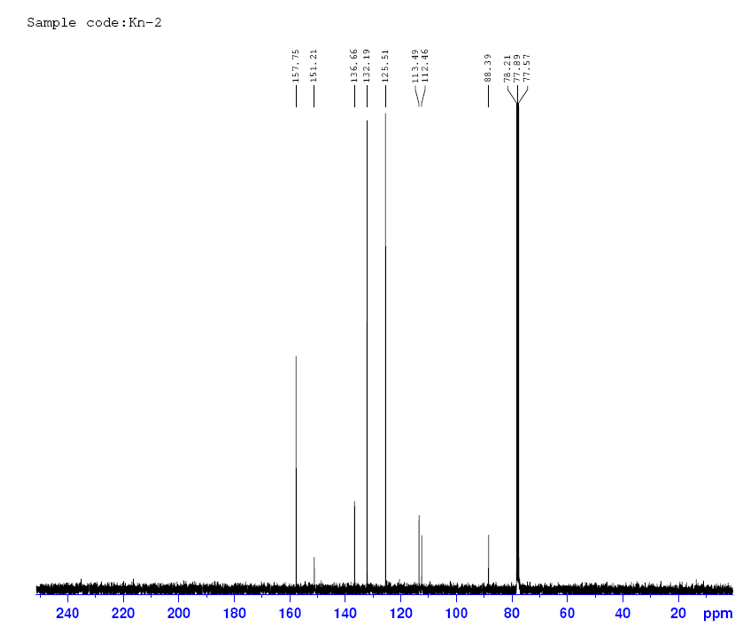


Figure S2. ^13^CNMR spectrum of 2-(4-nitrobenzylidene)malononitrile


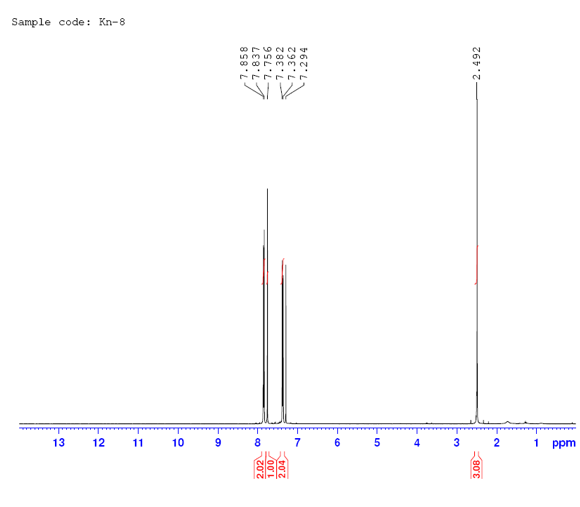


Figure S3. ^1^HNMR spectrum of 2-(4-methylbenzylidene)malononitrile


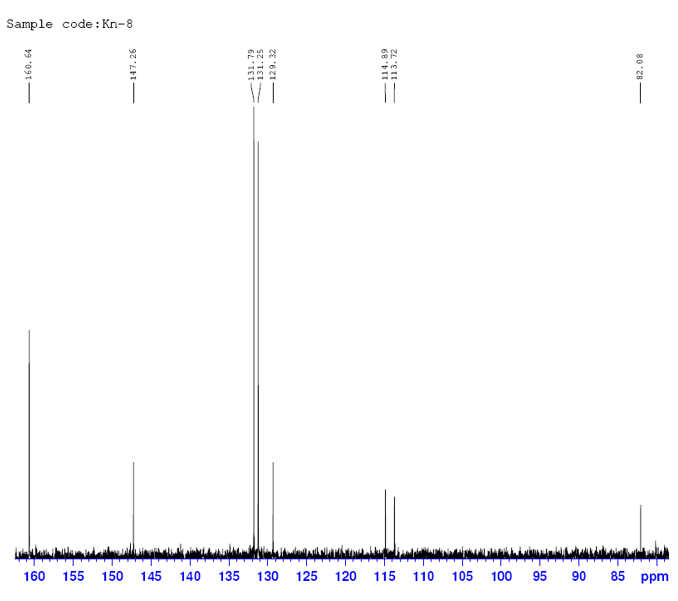


Figure S4. ^13^CNMR spectrum of 2-(4-methylbenzylidene)malononitrile


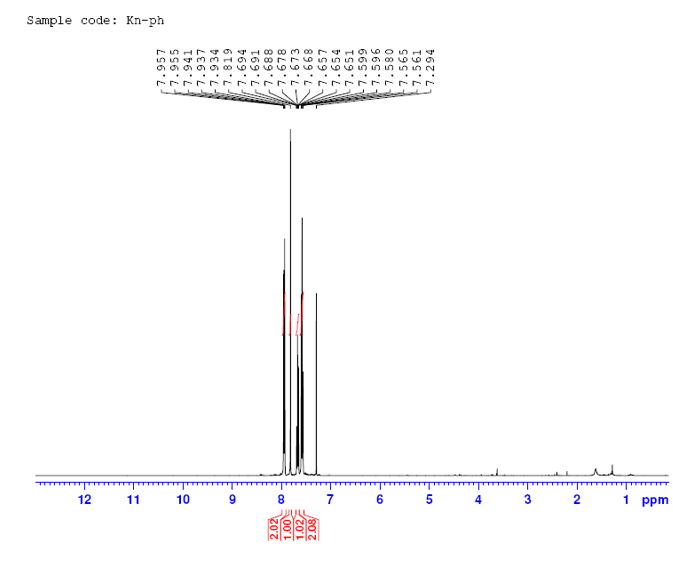


Figure S5. ^1^HNMR spectrum of 2-benzylidenemalononitrile


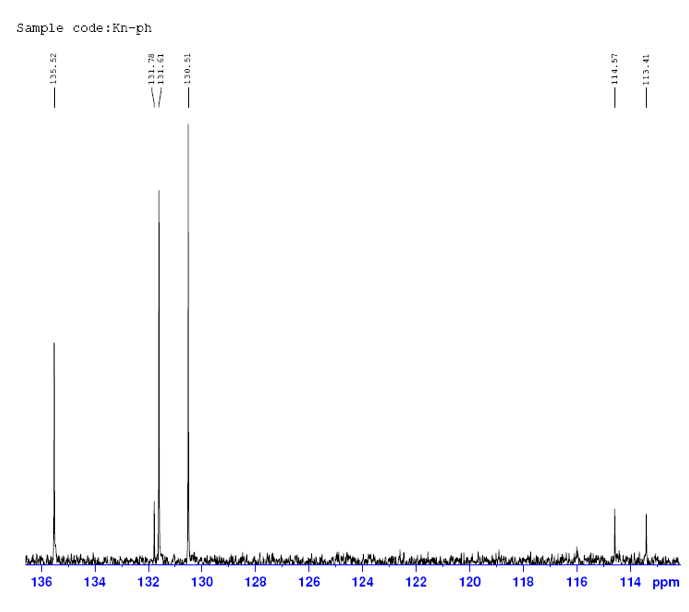


Figure S6. ^13^CNMR spectrum of 2-benzylidenemalononitrile


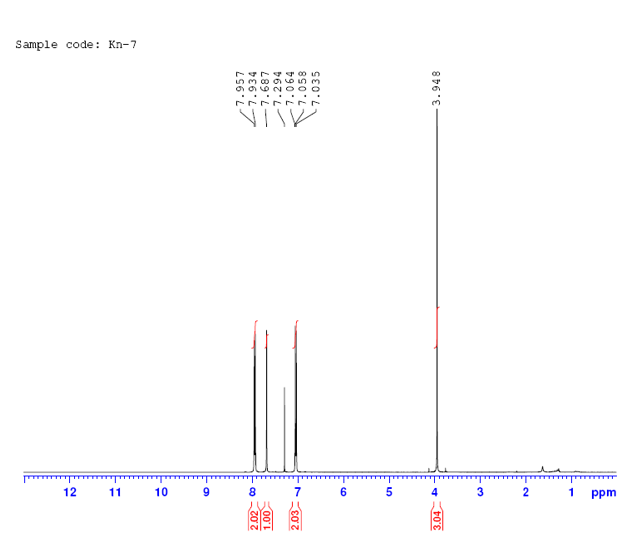


Figure S7. ^1^HNMR spectrum of 2-(4-methoxybenzylidene)malononitrile


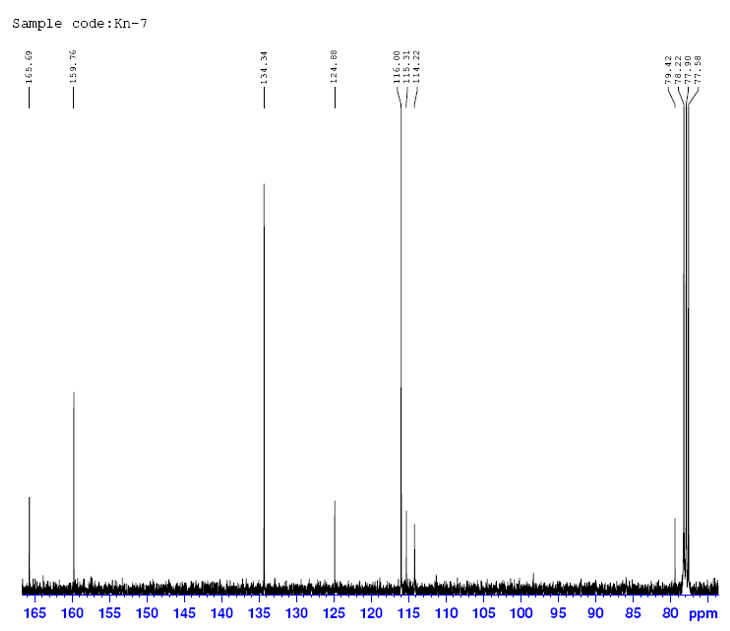


Figure S8. ^13^CNMR spectrum of 2-(4-methoxybenzylidene)malononitrile


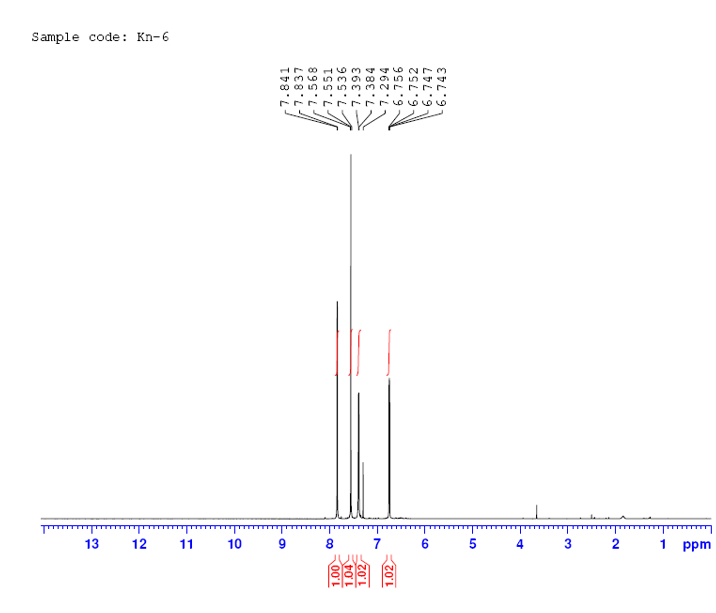


Figure S9. ^1^HNMR spectrum of 2-(furan-2-ylmethylene)malononitrile


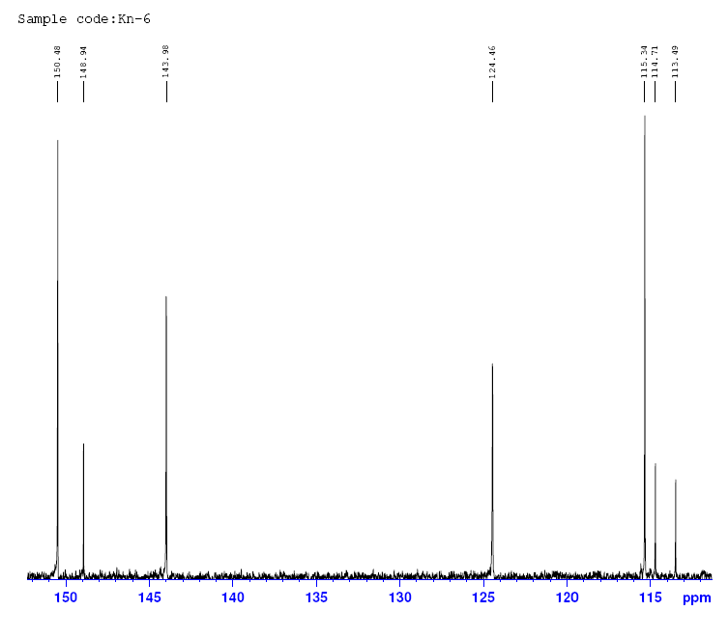


Figure S10. ^13^CNMR spectrum of 2-(furan-2-ylmethylene)malononitrile
